# A Lesson in Etiquette

**Published:** 1882-03

**Authors:** 


### A LESSON IN ETIQUETTE.

1. Always say, Yes, sir. No, sir. Thank you. If you please. Good night. Good morning. Never say "how?" instead of "what." Use no slang terms. Remember that good reading, good spelling and good grammar are the basis of a good education. Never call any one "Say," or "I say."

2. Clean faces, clean clothes, clean shoes, clean nails, and clean teeth indicate good breeding. Never leave your clothes about the room. Have a place for everything, and everything in its place.

3. Knock before entering a private room, and try to avoid leaving it with your back turned to the company. Never enter a private room or public place with your hat on.

4. Always offer your seat to a lady, or to an old gentleman. Let grown-up people and your visitors enter the carriage, the pew, or a room first; you can politely open the door and stand back to let them pass.

5. At table eat with your fork; sit up straight, never use your toothpick; do not fold your dinner napkin unless you are to remain for another meal, or unless you are at home. No one will wish to make use of a soiled dinner napkin. Do not scrape your plate, or mop it out with a piece of bread.

6. Never put your feet on cushions, chairs or tables.

7. Never overlook any one who is reading or writing. Never talk or read aloud when another is reading. When conversing listen attentively; never interrupt the conversation of others.

8. Never whisper in church or turn your head to see who is entering. Never whisper at public places, or in a private room, especially if any one else is singing or playing on the piano; at such times you should listen with polite attention.

9. Loud coughing, "hawking," yawning, sneezing and nose-blowing are ill-mannered. In every case cover your mouth with your handkerchief (which never examine—nothing is more vulgar except spitting on the floor).

10. Be careful not to injure the feelings of others by unkind remarks or smarting jests. Never tell tales; speak of others as if they were listening to you. Do not "make faces," "call names," or ridicule the poor or the unfortunate.

### GOLDEN RULES.

If you feel inclined to "tattle" (with grown-up people it is called gossip), first, think if what you are going to tell is *true;* secondly, if it is *kind;* and thirdly, if it is *necessary.* If not, you had better not tell it.

If you do make up your mind "to tell it," think of whom you tell it, to whom you tell it, and where you tell it.

People are generally more sorry that they have told such things than they are for being silent. It is said "speech is silver, silence is golden."

